# The strategic role of biotics in dental caries prevention: A scoping review

**DOI:** 10.1002/fsn3.4473

**Published:** 2024-09-26

**Authors:** Morteza Banakar, Gustavo Vicentis Oliveira Fernandes, Shahroo Etemad‐Moghadam, Roland Frankenberger, Maryam Pourhajibagher, Majid Mehran, Mohammad Hossein Yazdi, Roza Haghgoo, Mojgan Alaeddini

**Affiliations:** ^1^ Dental Research Center, Dentistry Research Institute Tehran University of Medical Sciences Tehran Iran; ^2^ Department of Pediatric Dentistry, Faculty of Dentistry Shahed University Tehran Iran; ^3^ A. T. Still University – Missouri School of Dentistry & Oral Health St. Louis Missouri USA; ^4^ Department of Operative Dentistry and Endodontics, Dental School University of Marburg and University Medical Center Giessen and Marburg Marburg Germany; ^5^ Biotechnology Research Center Tehran University of Medical Sciences Tehran Iran

**Keywords:** cariogenic bacteria, dental caries, oral microbiota, prebiotic, probiotic, symbiotic

## Abstract

Dental caries is a global oral health issue that is prevalent and preventable. Biotics (probiotics, prebiotics, symbiotics, and postbiotics) are recommended as low‐cost methods for preventing dental caries. This scoping review aimed to critically review the scientific evidence concerning the role of biotics in caries prevention and maintaining oral health benefits. A systematic search was conducted in several databases from 2012 onward, using specific keywords. The search resulted in 69 articles. While there is limited research on the mechanism of biotics in preventing caries, numerous studies have investigated the impacts of probiotics on decreasing caries risk factors. Probiotics can reduce cariogenic bacteria, reduce acidogenic bacteria, increase pH, and produce antimicrobial compounds. Probiotics can be used as a therapeutic approach to manage caries by restoring eubiosis at the host–microbial interface, which may not be accomplished with traditional therapies. Its positive effect on reducing dental caries is influenced by the choice of potent probiotic strains, appropriate dosage, treatment period, vehicle, and microbial interaction with the host. Specific oral bacteria have also been shown to utilize prebiotics such as urea and arginine, increasing pH levels. This highlights the potential of combining prebiotic and probiotic bacteria for caries prevention. In addition, this review is focused on bacterial‐derived compounds, namely postbiotics, due to their valuable effects in preventing caries. Biotics have demonstrated potential in preventing dental caries and maintaining oral health. Further research is needed to optimize their use and explore the potential of postbiotics for caries prevention.

## Graphical abstract

Probiotics can be used as a therapeutic approach to manage caries by restoring eubiosis at the host–microbial interface. Its positive effect depends on the potent probiotic strains, appropriate dosage, treatment period, vehicle, and microbial–host interaction. Specific oral bacteria have also been shown to utilize prebiotics such as urea and arginine, increasing pH levels. This highlighted the potential of mixing prebiotic and probiotic bacteria for caries prevention. Biotics have shown potential in preventing dental caries and maintaining oral health benefits.

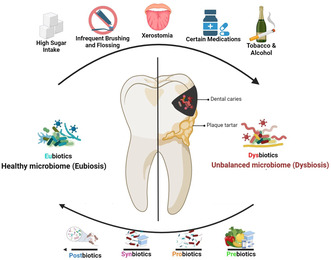

## 1 INTRODUCTION

Oral diseases pose a significant public health challenge worldwide, affecting individuals of all ages and stages of life. Dental caries, gingivitis, periodontal disease, and cancer are among the most prevalent oral health disorders (Sudha et al., 2020). Dental caries is the most common oral health concern, affecting an estimated 3.5 billion individuals globally, and in industrialized countries, it affects 60%–90% of children and adults (Petersen et al., 2020). The eubiotic state of host–microbe interactions in the oral cavity, a complex and dynamic ecosystem of microorganisms that interact symbiotically with their human host, is essential for maintaining host health. However, this state can shift to a pathogenic dysbiotic state for various reasons (Radaic & Kapila, 2021).

**FIGURE 1:**
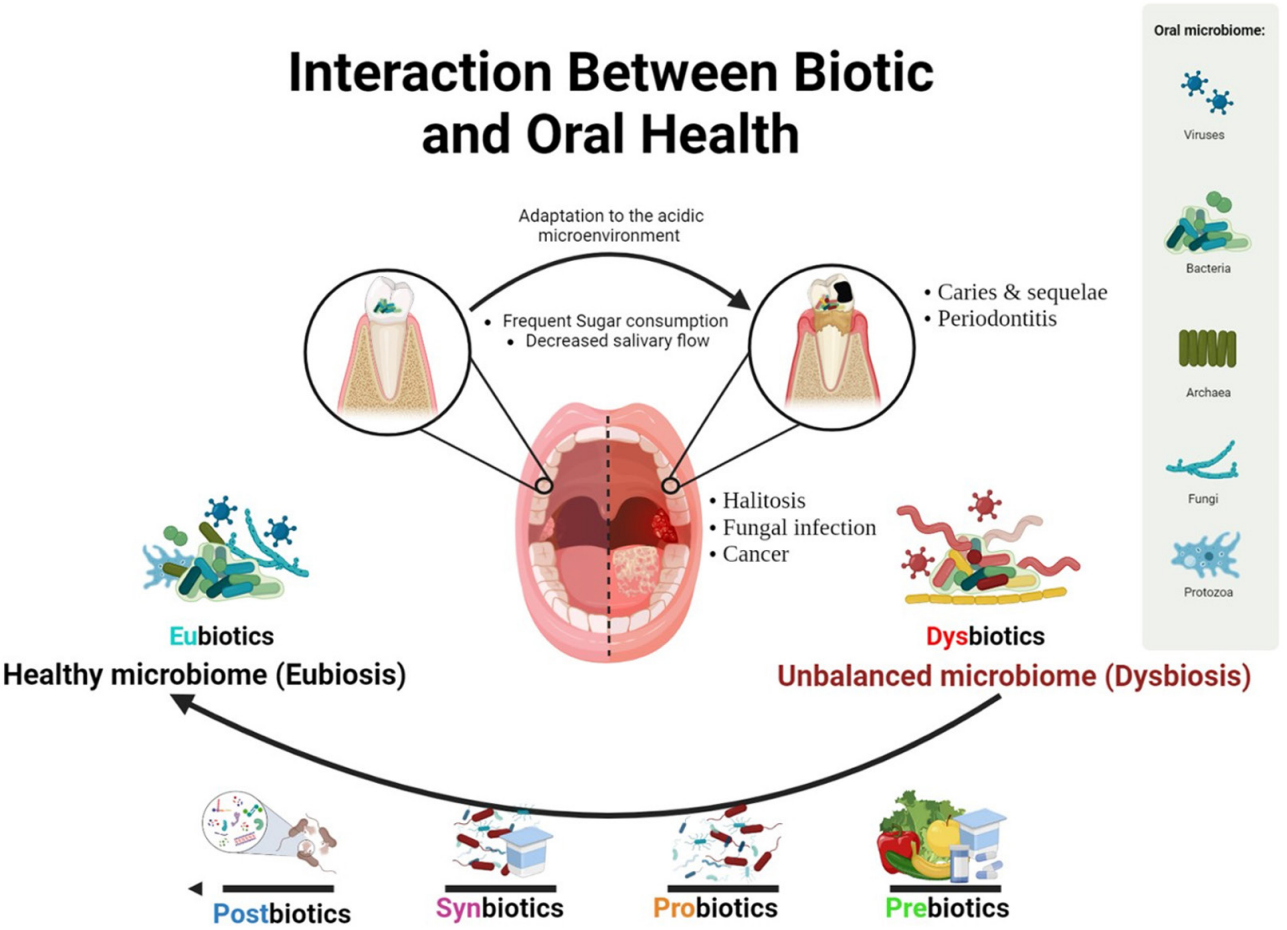
Summary of interactions between biotics and oral health.

**FIGURE 2:**
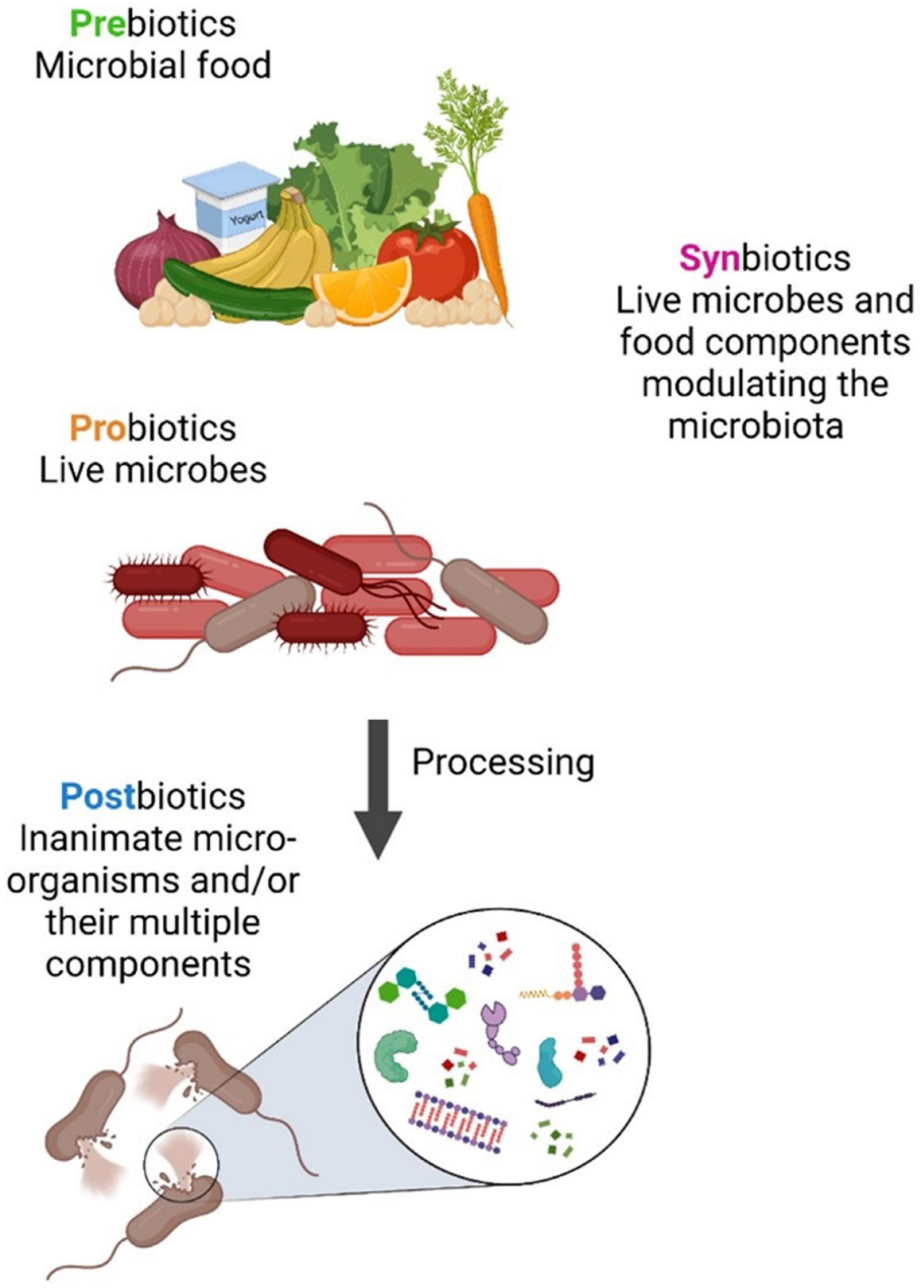
Prebiotics, probiotics, synbiotics, and postbiotics (Favero et al., 2022).

The oral microbiota comprises up to a thousand different species of microorganisms, including bacteria, fungi, viruses, archaea, and protozoa, which interact with the host to create a dynamic ecology (Figure 1). The development of caries is influenced by multiple factors, including host genetic predisposition, inadequate oral hygiene, smoking habits, dietary choices, systemic health conditions, and decreased saliva production. Caries arise due to dysbiosis of the oral microbiome (Chen et al., 2021; Chen, Daliri, et al., 2020). While antimicrobial substances such as fluoride, chlorhexidine, triclosan, and antibiotics are often used to manage oral diseases, their use has been associated with increasing antimicrobial resistance and side effects, and they can also eliminate eubiotic microbes along with the dysbiotic ones (Marinho et al., 2022; Radaic & Kapila, 2021). Hence, alternative treatments focusing on restoring the oral microbiota equilibrium are required. Then, biotic supplementation, which encompasses prebiotics, probiotics, synbiotics, and postbiotics, is a promising strategy for preventing and treating oral diseases (Figure 2). This growing interest in biotics is reflected in the expanding global market for these products, particularly in oral health.

The global biotic market, particularly for probiotics, has experienced substantial growth in recent years. According to a report by Allied Market Research, the global oral care probiotic market was valued at $1.2 billion in 2020 and is projected to reach $2.3 billion by 2028, growing at a CAGR (Compound Annual Growth Rate) of 8.9% from 2021 to 2028 (Markets and Markets – Global Forecast to 2029). This growing interest in biotics for oral health can be attributed to several factors, including increasing consumer awareness of the limitations of traditional oral care approaches, a rising preference for preventative oral health management, and emerging scientific evidence supporting the role of beneficial microbes in maintaining oral homeostasis and preventing diseases like dental caries, periodontal disease, and halitosis (Inchingolo et al., 2023; Laleman et al., 2021). Developing novel biotic‐based products that offer convenient and effective ways for consumers to incorporate these ingredients into their daily oral hygiene routines further drives market expansion. As the global population becomes more conscious of oral health's importance and biotics' potential to address unmet needs, the market for these products is expected to continue its rapid expansion in the coming years (Innova Market Insights, 2023; Precision Business Insights, 2024).

Probiotics are defined as live bacteria with beneficial effects on the health of the host organism and have a regulatory effect on the host immune system via keeping microbial equilibrium (Allaker & Douglas, 2015; Allaker & Stephen, 2017; Fuad et al., 2023; Inchingolo et al., 2023). They have been shown to form a biofilm that acts as a protective layer for oral tissues, preventing bacterial infections by filling the areas where pathogens would penetrate (Allaker & Douglas, 2015; Allaker & Stephen, 2017; Fuad et al., 2023; Meyer et al., 2021). Prebiotics, a selectively utilized substrate by host microorganisms, confer health benefits, shift the microflora balance, and stimulate the development and activity of favorable probiotic organisms, improving host health (Agarwal et al., 2022; Palai et al., 2020; You et al., 2022). Synbiotics, a combination of probiotics and prebiotics, may be more effective than either alone in preventing dental caries due to their synergistic effects. However, there is little evidence for this (Bijle, Ekambaram, et al., 2020; Kozak & Pawlik, 2023). The International Scientific Association for Probiotics and Prebiotics (ISAPP) has revised the definition of “postbiotics” as of 2021 to include inactivated probiotic cells and/or their components that still maintain the live form's health benefits to the host (Salminen et al., 2021). They are non‐viable microorganisms or bioactive compounds released by or created through the activity of probiotic microbes, including cell‐free supernatants, bacteriocins, organic acids, secreted proteins, and biosurfactants. Postbiotics have shown antimicrobial activity against the microbial etiology of dental caries (Banakar et al., 2023; Giordani et al., 2021; Rad et al., 2023; Vinderola et al., 2022). However, using postbiotics to prevent oral diseases, especially dental caries, is a new concept, and few studies have reviewed their efficacy (Rad et al., 2023). In this scoping review, we aim to evaluate the role of biotics in dental caries prevention, the data supporting their bioactivities, and the mechanisms behind their beneficial effects on the prevention of dental caries.

## 2 MATERIALS AND METHODS

### 2.1 Study protocol

The study protocol was developed using the PRISMA‐ScR guidelines (Tricco et al., 2018), clearly stating the study's purpose, search strategy, inclusion and exclusion criteria, data extraction, and synthesis methods. Data summary charting form were also developed to extract relevant information from the included studies.

### 2.2 Literature search

A comprehensive literature search was conducted using EMBASE, MEDLINE/PubMed, Google Scholar, Web of Science, Cochrane, and Scopus databases. The search strategy was developed using MeSH, Emtree, and free‐text terms related to dental caries, probiotics, prebiotics, symbiotics, and postbiotics. The search terms used were “probiotic” OR “prebiotic” OR “symbiotic” OR “postbiotic” AND “dental caries” OR “tooth decay” OR “cariogenic bacteria” OR “*Streptococcus mutans*” OR “*lactobacilli*”. The search was limited to articles published in English since 2012. In addition, a manual search was conducted to include gray literature sources, such as theses, conference proceedings, organizational reports, websites, and unpublished research and data.

### 2.3 Eligibility criteria

Studies were included in the scoping review if they investigated the role of probiotics, prebiotics, symbiotics, or postbiotics in preventing dental caries. The study designs included in vitro investigations, clinical trials, systematic reviews, meta‐analyses, umbrella reviews, and narrative reviews. Only studies published in English were included. Commentaries, animal studies, opinions, and studies that did not have a keyword‐specific component were excluded. Studies examining biotics' effects on other oral diseases or periodontitis were also excluded.

### 2.4 Study selection

After removing duplicates using EndNote software, two reviewers independently screened the titles and abstracts of the identified studies for eligibility. Full texts of potentially eligible articles were then reviewed to determine their inclusion in the scoping review. Any reviewer disagreements were resolved through discussion and, if necessary, consultation with a third reviewer. A Preferred Reporting Items for Systematic Reviews and Meta‐Analyses (PRISMA) flow diagram was used to document the study selection process (Page et al., 2021).

## 3 RESULTS

### 3.1 Literature search results

**FIGURE 3:**
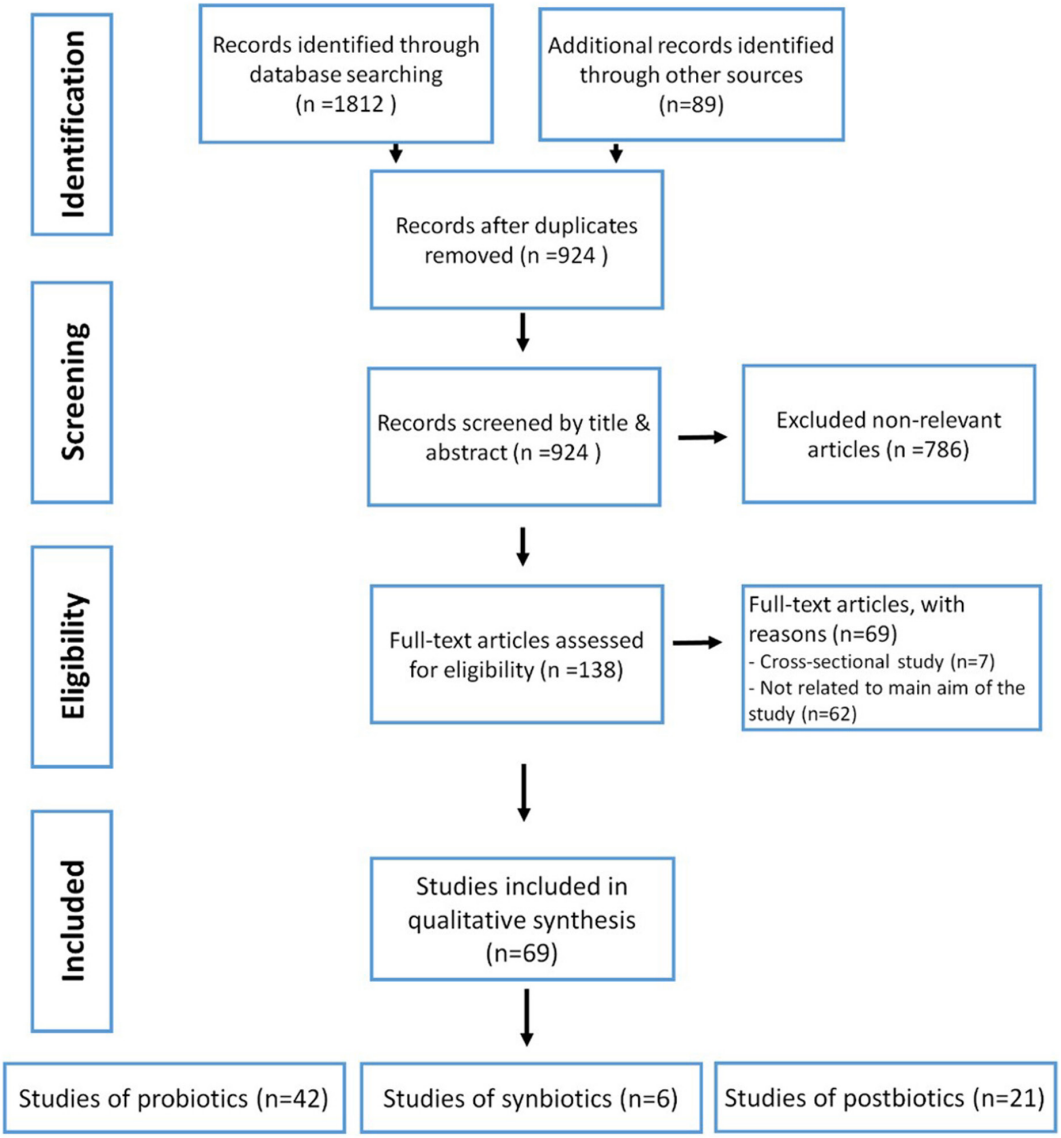
Flow diagram of screened records.

The data‐gathering process for this review is presented in Figure 3. A total of 1812 records were found, with an additional 89 studies located through a citation search. After screening and eligibility assessments, 138 full‐text articles were evaluated, of which 69 met the inclusion and exclusion criteria and were included in this review. Of the included studies, 42 articles assessed the effects of probiotics, 21 examined postbiotics, and 6 evaluated synbiotics in preventing dental caries. In interpreting the results, prebiotics and synbiotics were combined for brevity, and only those prebiotics whose probiotics were mentioned in the relevant study were included.

### 3.2 Interaction between oral microbiome and dental caries

**FIGURE 4:**
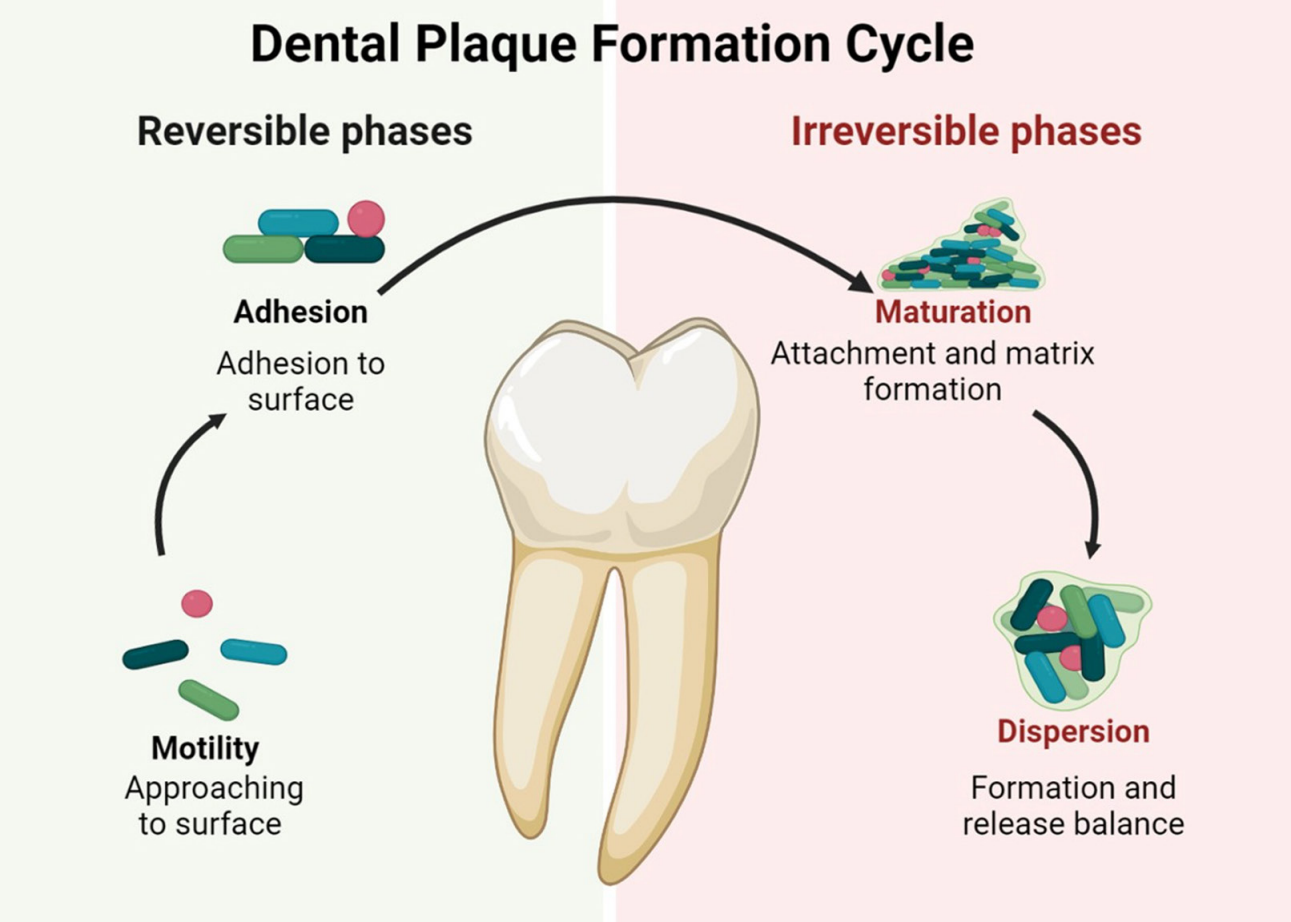
Stages of dental biofilm (plaque) formation.

The oral microbiome plays a crucial role in the host's health and disease condition, with interspecies and host–microbe interactions significantly affecting the microbial composition (Kilian et al., 2016; Radaic & Kapila, 2021). The oral biofilm, which contains most of the oral microbiome, is a dynamic bacterial population that can survive in low‐pH environments due to its quick metabolism of sucrose, fructose, and glucose. Early colonizers adhere to enamel and periodontal tissue, forming biofilms in the mouth (Figure 4). The extracellular polymeric matrix (EPM), including polysaccharides, proteins, lipids, and extracellular DNA, is produced and released by surface‐attached bacteria after colonization and provides bacterial nutrition. The EPM makes bacteria in the biofilm 1000 times more resistant to antibiotics than planktonic bacteria, making it more challenging for antibiotics to penetrate the biofilm (Topka‐Bielecka et al., 2021).

**FIGURE 5:**
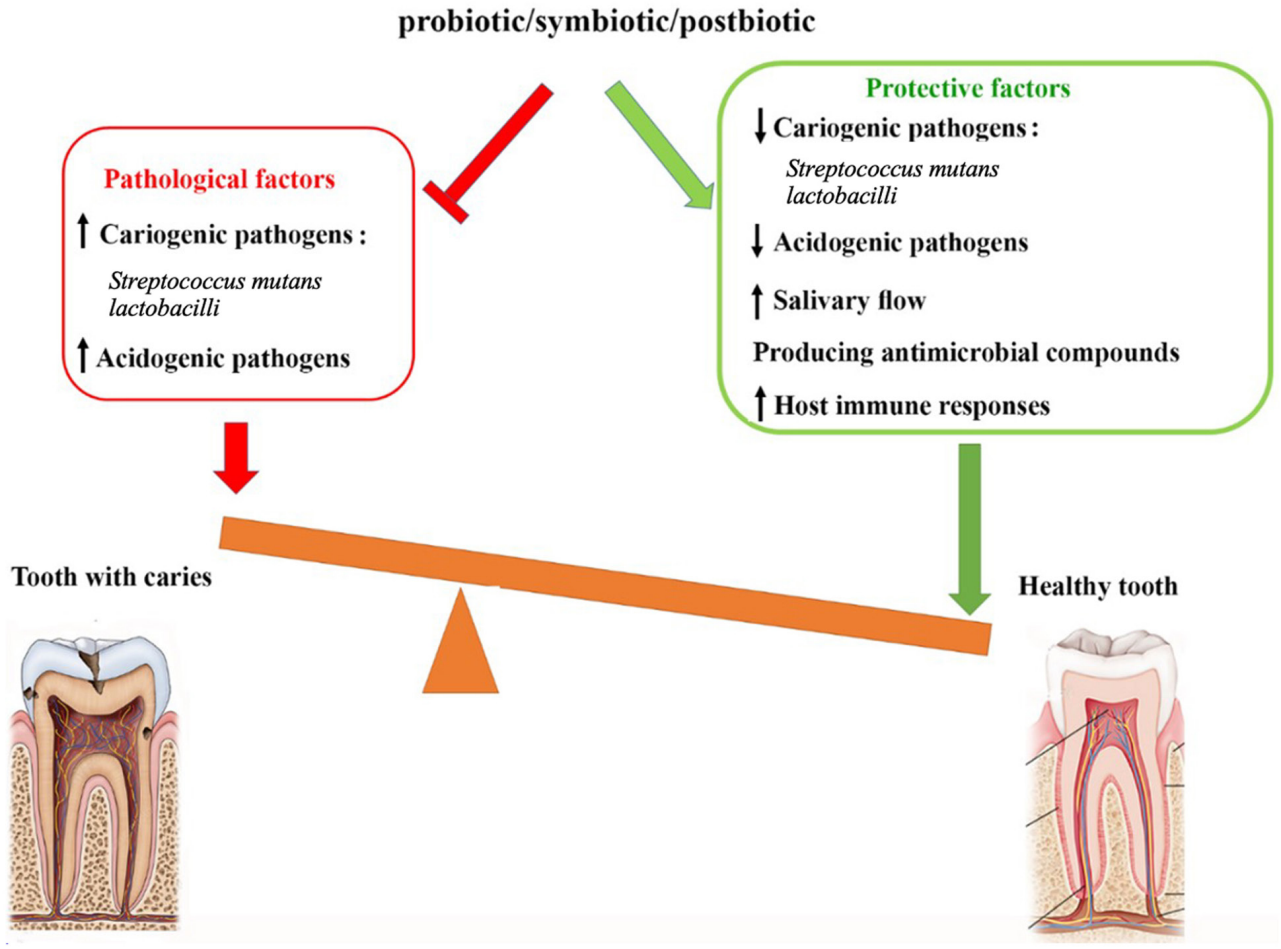
Role of biotics in dental caries prevention.

Oral biofilms contain many different types of microbes, some entirely non‐pathogenic, while others have pathogenic potential. According to the “Ecological Plaque Hypothesis,” in a healthy environment, all microorganisms live in harmony with one another and the host (Radaic & Kapila, 2021). However, regular sugar consumption promotes the substitution of aciduric bacterial species, particularly *Streptococcus*, *Lactobacillus*, and *Actinomycetes*, which are associated with an increased risk of caries (Giordani et al., 2021). The acid these bacteria produce eventually demineralizes the hydroxyapatite crystal, causing caries. Remineralization occurs after removing acidic residues (Valm, 2019; Zhang et al., 2022). *Streptococcus mutans* is one of the principal pathogens implicated in caries, and *Lactobacillus* may be considered another significant cariogenic bacterium in the oral flora after *S. mutans* (Ahirwar et al., 2019; Mallya & Mallya, 2020). Biotics, including probiotics, postbioics, and synbiotics, have recently emerged as a promising new approach to managing dental caries (Amargianitakis et al., 2021; Voidarou et al., 2020). Figure 5 briefly describes the role and mechanism of biotics in preventing dental caries.

### 3.3 The role of probiotics in dental caries

Meurman et al. (1994) were the first to introduce probiotics into dental clinical practice. Probiotics have been shown to effectively control the growth and proliferation of dysbiotic bacteria, especially cariogenic bacteria, thereby significantly preventing dental caries. The mechanisms by which probiotics modify the oral environment and prevent dental caries can be categorized into four main categories:

1. *Competitive exclusion*: Probiotics can compete with pathogenic bacteria for adhesion sites and nutrients in the oral cavity. This competitive exclusion of pathogens is one of the main proposed mechanisms by which probiotics prevent dental caries (Allaker & Stephen, 2017; Devine et al., 2015; Lin et al., 2018; Shakib et al., 2020). Probiotic microorganisms can also alter the salivary pellicle or excrete specific biosurfactants that prevent pathogenic bacteria from adhering (Amargianitakis et al., 2021; Sharma et al., 2016).
2. *Production of antimicrobial compounds*: Probiotics can produce various antimicrobial compounds that inhibit the growth of cariogenic bacteria. These compounds include hydrogen peroxide, organic acids, fatty acids, and bacteriocins (Di Pierro et al., 2015; López‐López et al., 2017). For example, *Lactobacillus Gorbach‐Goldin* (LGG), a commonly used probiotic strain, produces an antibacterial compound with a wide range of activity against both Gram‐positive and Gram‐negative bacteria (Jenab et al., 2020). Reuterin and reutericyclin, both produced by *Lactobacillus reuteri*, have antibacterial effects on target cells by generating oxidative stress and modifying the transmembrane (Monika et al., 2021).
3. *Modulation of the immune system*: Probiotics can stimulate and modulate the host immune response by increasing epithelial barrier function and influencing innate and adaptive immune responses (Diaz et al., 2012; Schincaglia et al., 2017). Probiotics have been shown to enhance the phagocytic activity of macrophages, neutrophils, and natural killer cells and interact with host mucosal cells through their extracellular products (Amargianitakis et al., 2021; Delcenserie et al., 2008). For instance, *Bifidobacterium lactis*, *L. rhamnosus*, and *Lactobacillus acidophilus* improve leukocyte phagocytic capability and elevate serum IgA levels (Ericson et al., 2013; Pahumunto et al., 2019; Teughels et al., 2011).
4. *Alteration of the oral biofilm composition*: Probiotics can modulate the oral biofilm composition through pathogen antagonistic interactions and co‐aggregation mechanisms, which reduces the potential pathogen load in oral biofilm, biofilm bacteria' pathogenicity, and cariogenic potential (Sudha et al., 2020; Taheur et al., 2016). Probiotic *lactobacilli* can co‐aggregate with *S. mutans* and other caries‐associated bacteria, inhibiting the development of *S. mutans* (Hasslöf et al., 2013).
5. *Modulation of oral pH and acidogenicity*: Cariogenic bacteria thrive in acidic environments and produce acids that demineralize tooth enamel. Probiotics can directly inhibit the metabolic pathways of cariogenic bacteria, reducing their acid production and mitigating their cariogenic potential. Some probiotic strains can produce base compounds that neutralize the acids produced by cariogenic bacteria, thereby increasing oral pH and reducing the risk of enamel demineralization (Campus et al., 2014; Di Pierro, 2016; Ferrer et al., 2020; Lai et al., 2021; Lin et al., 2017; López‐López et al., 2017; Saha et al., 2014).

Probiotic strains from the *Streptococcus*, *Lactobacillus*, and *Bifidobacterium* genera are the most often studied for dental caries (Devine & Marsh, 2009) (Table 1). Research indicates that probiotic supplementation can improve oral health and reduce the risk of developing caries by reducing the count of *S. mutans* and *Lactobacillus* in saliva or plaque (Alamoudi et al., 2018; Burton, Drummond, et al., 2013; Burton, Wescombe, et al., 2013; Campus et al., 2014; Cannon et al., 2013; Di Pierro et al., 2015; Ferrer et al., 2020; Ghasemi et al., 2017; Gizani et al., 2016; Jose et al., 2013; Juneja & Kakade, 2012; Kaur et al., 2018; Lai et al., 2021; Lin & Pan, 2014; Mahantesha et al., 2015; Manmontri et al., 2020; Pahumunto et al., 2018; Saha et al., 2014; Sudha et al., 2020; Teanpaisan & Piwat, 2014; Villavicencio et al., 2018; Wasfi et al., 2018; Yadav et al., 2015; Zare et al., 2015). Sakhare et al. (2021) have shown that long‐term (21 days) administration of probiotics *L. acidophilus* (La5) and *B. lactis* (Bb12) may play a preventive role against caries by reducing the number of cariogenic bacteria. In contrast, short‐term (7 days) management does not show the same effect. In some other studies, we have seen the beneficial effects of *B. lactis* (Bb12) and *L. acidophilus* (La5) in reducing *S. mutans* levels with increasing the study period (Ashwin et al., 2015; Mahantesha et al., 2015). The effectiveness of probiotics in inhibiting *S. mutans* growth and plaque formation, as well as generating an acidic environment or bacteriocin‐like polypeptides, was dependent on the specific strains of bacteria used (Lin et al., 2015; Schwendicke et al., 2017).

**TABLE 1:** Probiotic strains have been studied for dental caries in vitro and in vivo.

References	Subjects/sample	Probiotics strain	Vehicle	Dose	Duration	Effects
Sakhare et al. (2021)	Children (6–12 years old; *n* = 62)	*Lactobacillus acidophilus* (La5) *Bifidobacterium lactis* Bb12	Curd (Amul's Probiotic Dahi)	100 g curd (1 × 10^6^ bacterial count/mL), twice daily	7 days	‐ Salivary pH ‐ Salivary *Streptococcus mutans* count
					3 weeks	Salivary pH ↓ *S. mutans* count
Burton, Drummond, et al. (2013), Burton, Wescombe, et al. (2013)	Children (5–10 years old; *n* = 40)	*Streptococcus salivarius* M18	Probiora3® Lozenge	2 tablet/day	3 months	↓Cariogenic bacteria count ↓ *S. mutans* count
Cannon et al. (2013)	Children (6–12 years old; *n* = 60)	*Streptococcus uberis* KJ2TM *Streptococcus oralis* KJ3TM *Streptococcus rattus* JH145TM (EvoraKids)	Chewable tablet	≥100 million CFU, 1 per day	30 days	↓ Salivary *S. mutans* count ↓ Salivary *lactobacilli* count
		*Lactobacilli reuteri* (PerioBalance)	Lozenges	200 million CFU, 1 per day	28 days	↓ Salivary *S. mutans* count ↓ Salivary *lactobacilli* count
Campus et al. (2014)	Children (6–8 years old; *n* = 191)	*Lactobacillus brevis* CD2	Lozenges	2 × 10^9^ CFU, twice a day	6 weeks	↓ *S. mutans* count, ↑ plaque pH ↓ gingival bleeding value
Stensson et al. (2014)	Children (9 years old; *n* = 113)	*L. reuteri* DSM 55730	Drops	10^8^ CFU/mL, 65 mL/day	1 year	↓ Gingival Bleeding ‐ Salivary *S. mutans* count ‐ Salivary *lactobacilli* count ‐ Salivary secretory IgA ‐ Plaque index
Ashwin et al. (2015)	Children (6–12 years old; *n* = 60)	*B. lactis* Bb‐12 *Lactobacillus acidophilus* La‐5	Ice cream	1 × 10^6^ CFU	7 days/30 days	↓ *S. mutans* count
					6 months	*S. mutans* count
Rodríguez et al. (2016)	Children (2–3 years old; *n* = 261)	*Lactobacillus rhamnosus* SP1	Milk	150 mL milk per day, 10^7^ CFU/mL	10 months	↓ Dental caries
Hedayati‐Hajikand et al. (2015)	Children (2–3 years old; *n* = 138)	*S. uberis* KJ *S. oralis* KJ *S. rattus* JH14	Chewing Tablet (ProBiora3®)	1 × 10^8^ CFU	12 months	↓ Dental caries
Cildir et al. (2012)	Children (4–12 years old; *n* = 19)	*L. reuteri* DSM 17938 *L. reuteri* ATCC PTA 5289	Drops	1 × 10^8^ CFU/5 drops	25 days	‐ Salivary *S. mutans* count ‐ Salivary *lactobacilli* count
Mahantesha et al. (2015)	Children (6–12 years old; *n* = 50)	*B. lactis* Bb‐12 and *L. acidophilus* La‐5 (Amul®)	Ice cream	1 × 10^6^ CFU	7 days	↓ *S. mutans* count
					90 days	↓ *S. mutans* count
		*Lactobacillus casei*	Drink (Yakult)	6.5 billion CFU	7 days	↓ *S. mutans* count
					90 days	‐ *S. mutans* count
Kaur et al. (2018)	Children (7–12 years old; *n* = 40)	*L. reuteri* DSM 17938 (BioGaia™ ProDentis) *L. reuteri* ATCC PTA 5289 (*L. reuteri* Prodentis)	Chewing gums	Three gums per day for 20 min	3‐week	↓ Plaque index ↓ Gingival index ↓ *S. mutans* count
Villavicencio et al. (2018)	Children (1–15 years old; *n* = 321)	*L. rhamnosus*	Milk (200 mL/day)	5 × 10^6^ CFU/mL	9 months	‐ *S. mutans* count ↓ *lactobacilli* count ‐ Dental caries ‐ Dental plaque and pH ↑ Salivary buffer capacity
		*Bifidobacteruim longum*		3 × 10^6^ CFU/mL		
Angarita‐Díaz et al. (2020)	Children (2–3 years old; *n* = 261)	*L. rhamnosus* GG	Milk (200 mL/day)	7.5 × 10^5^ CFU/mL	3 months	‐ *S. mutans* count ↓ Salivary pH ‐ Dental plaque
		*B. longum*		4.5 × 10^5^ CFU/mL		
Sudha et al. (2020)	Children (5–15 years old; *n* = 48)	*Bifidobacteruim coagulans* Unique IS2	Chewable tablets	2 billion CFU/day	2 weeks	↓ Salivary *S. mutans* count ↓ Plaque *S. mutans* count ↓ Salivary *lactobacilli* count ↓ Plaque *lactobacilli* count ‐ Salivary pH ‐ Plaque pH
Di Pierro (2016)	–	*S. salivarius* M18 (Carioblis®)	Tablet	10^6^–10^9^ CFU	28 days	↓Gingival inflammation ↓ Plaque levels ↑Oral pH ↓ Dental caries
Di Pierro et al. (2015)	Children (6–17 years old; *n* = 76)	*S. salivarius* M18 (Carioblis®)	Tablet	1 billion CFU/day	90 days	↓ *S. mutans* count ↓ Plaque levels ↓ Dental caries ↓ Global Cariogram outcome
Taipale et al. (2013)	Children (4 years old; *n* = 106)	*Bifidobacterium animalis* subsp. *lactis* BB‐12	Tablet	10^10^ CFU/mL, twice daily	22–23 months	‐ Dental caries
Lin et al. (2017)	Children (7–11 years old; *n* = 18)	*L. casei shirota*	Drink (Yakult)	3.6 × 10^9^ CFU/day	7 days	↑ Plaque pH ‐ Salivary *S. mutans* count ‐ Salivary *lactobacilli* count
Hasslöf et al. (2013)	Children (9 years old; *n* = 179)	*Lactobacillus paracasei* F19	Cereals	10^8^ CFU/day	9 months	‐ Salivary *S. mutans* count ‐ Salivary *lactobacilli* count ‐ Dental caries
Juneja and Kakade (2012)	Children (12–15 years old; *n* = 40)	*L. rhamnosus* hct 70	Milk	2.34 × 10^9^ CFU/day, twice daily	3 weeks	↓ *S. mutans* count ↓ Dental caries
Yadav et al. (2015)	Children (6–8 years old; *n* = 62)	*L. casei shirota*	Milk	10 mL/day	10 days	↓ *S. mutans* count
Aminabadi et al. (2011)	Children (6–12 years old; *n* = 105)	*L. rhamnosus* GG	Yogurts (15–20 mL/day)	2 × 10^8^ CFU/g	3 weeks	‐ *S. mutans* count
Pahumunto et al. (2018)	Children (1.5–5 years old; *n* = 124)	*L. paracasei* SD1	Milk powder (5 g/day)	1 × 10^7^ CFU/g	3 months	↓ *S. mutans* count
Alamoudi et al. (2018)	Children (3–6 years old; *n* = 178)	*L. reuteri* DSM 17938 *L. reuteri* ATCC PTA 5289	Lozenges	≥2 × 10^8^ total CFU/lozenge, twice daily	28 days	↓ Salivary *S. mutans* count ↓ Salivary *lactobacilli* count ‐ Salivary buffering capacity ↓ plaque accumulation
Manmontri et al. (2020)	Children (1–5 years old; *n* = 354)	*L. paracasei* SD1	Milk powder (3 g/day)	1.8 × 10^7^ CFU/mL	6 months	↓ Salivary *S. mutans* count ↓ plaque *S. mutans* count ↑ Salivary *lactobacilli* count ↑ plaque *lactobacilli* count
Lai et al. (2021)	Children (4–14 years old; *n* = 68)	*L. brevis* CD2	Lozenge	2 × 10^9^ CFU/lozenge	60 days	↓ Salivary *S. mutans* count ↑ Plaque pH ↓ Bleeding score
Jose et al. (2013)	Orthodontic Adults (14–29 years old; *n* = 60)	–	Curd (Active Plus) GD Probiotic Toothpaste	200 mg/day, twice daily	30 days 30 days	↓ Plaque *S. mutans* count ↓ Plaque S. *mutans* count
Nishihara et al. (2014)	Adults (22–26 years old; *n* = 64)	*L. salivarius* WB21, *L. salivarius* TI 2711	Tablet	2.0 × 10^9^ CFU/day	2 weeks	‐ *S. mutans* count ↑ *lactobacilli* count ‐ Salivary flow ‐ Salivary pH ↑ Salivary buffering capacity
Teanpaisan and Piwat (2014)	Adults (18–25 years old; *n* = 40)	*L. paracasei* SD1	Milk powder	7.5 × 10^9^ CFU/day	4 weeks	↓ Salivary *S. mutans* count ‐ Salivary yeast counts ↑ Salivary *lactobacilli* count
Pinto et al. (2014)	Adults orthodontic patients (10–30 years old; *n* = 30)	*B. animalis lactis* DN‐173010	Yogurts (200 g/day)	–	1‐week OR 2‐week (4‐week washout period)	‐ Salivary *S. mutans* count ‐ Salivary *lactobacilli* count ‐ Plaque *S. mutans* count ‐ Plaque *lactobacilli* count ‐ Salivary total cultivable microorganism counts ↓ Plaque total cultivable microorganism counts
Laleman et al. (2015)	Adults (37–58 years old; *n* = 48)	*S. oralis* KJ3, *S. uberis* KJ2 and *S. rattus* JH145	Tablet	10^8^ CFU of each strain/tablet, twice a day	12 weeks	↓ Probing pocket depth Improvement in clinical attachment level, gingival recession, bleeding, gingival indexes ↓ Salivary *Prevotella intermedia* counts
					24 weeks	↓ Probing pocket depth Improvement in clinical attachment level, gingival recession, bleeding, gingival indexes ‐ Salivary *P. intermedia* counts
Zare et al. (2015)	Adults (18–30 years old; *n* = 66)	*B. lactis*	Yogurt (300 g/day)	10^6^ CFU/mL	2 weeks	↓ *S. mutans* count ↓ *lactobacilli* count
Gizani et al. (2016)	Adolescents (mean age 15.9 years; *n* = 85)	*L. reuteri* DSM 17938	Lozenges	Once a day, 1 × 10^8^ CFU/tablet	17 months	‐ *S. mutans* count ↓ *lactobacilli* count ‐ Incidence of white spot lesion
		*L. reuteri* ATCC PTA 5289	Lozenges	Once a day, 1 × 10^8^ CFU/tablet	17 months	
Ghasemi et al. (2017)	Adolescents (19–27 years old; *n* = 50)	*L. acidophilus* ATCC 4356 *Bifidobacterium bifidum* ATCC 29521	Yogurt (200/day)	1.5 × 10^8^ CFU/g	3 weeks	↓ *S. mutans* count
Koopaie et al. (2019)	Adults (mean age 41.67 years; *n* = 40)	*Bifidobacterium coagulans*	Cake (70 g/day)	–	1 weeks	‐ *S. mutans* count ‐ Salivary pH
Ferrer et al. (2020)	Adults (25–35 years old; *n* = 11)	*Streptococcus dentisani* CECT7746	Vials	10^10^ CFU/vial	Single‐dose: (5 min) Multi‐dose: 7 days	↓ *S. mutans* count ↑ Salivary pH
Javid et al. (2020)	Adults (18–30 years old; *n* = 66)	*B. lactis* Bb12	Yogurt	300 g/day	2 weeks	↓ Salivary *S. mutans* count ↓Salivary *lactobacilli* count
Schwendicke et al. (2014)	Bovine enamel and dentin samples (*n* = 240)	*L. rhamnosus* GG	Bacterial Culture	7 × 10 ^6^ CFU/mL, twice/day OR 6 times/day	10 days	↑ Mineral loss ↑ Dental caries ‐ *S. mutans* count
Saha et al. (2014)	*Streptococcus mutans*	*L. reuteri* NCIMB 701359, NCIMB 701089, NCIMB 702655 and NCIMB 702656	Bacterial cultures	10^8^ CFU/mL	24 h incubation	↓ *S. mutans* count ↑ Oral pH ↓NO production ↑Antioxidant production No bacteriocin
Wasfi et al. (2018)	*S. mutans*	*L. casei* subspecies *casei* (ATCC 393), *L. reuteri* (ATCC 23272), *L. plantarum* subspecies *plantarum* (ATCC 14917) and *L. salivarius* (ATCC 11741)	Bacterial cultures	–	Overnight incubation	↓ *S. mutans* count ↓ Cell adherence ↓ Acid tolerance genes (atpD and aguD genes), EPS‐producing genes (gtfBCD and sacB) and quorum‐sensing genes (vicKR and comCD) ↑ IFN‐γ ↓ IL‐10
López‐López et al. (2017)	*Streptococcus dentisani* 7746	*S. dentisani*	Bacterial cultures	6.5 × 10^8^ CFUs/mL	30 min incubation	↑ Inhibitors of peptidic nature (bacteriocins) ↓ Growth of *S. mutans* and *S. sobrinus* ↑ Buffers acidic pH
	*S. dentisani* 7747			3.9 × 10^8^ CFUs/mL		

Abbreviations: ‐, no effect; ↑, increase; ↓, decrease; CFU, colony‐forming units; *S. mutans*, *Streptococcus mutans*.

Studies have found no significant changes in the interspecies balance of the microbiota community in individuals who take probiotics (*Streptococcus salivarius* M18, LGG, and *B. lactis* BB‐12) (Burton, Drummond, et al., 2013; Burton, Wescombe, et al., 2013; Toiviainen et al., 2015). While most studies support the role of probiotics in caries prevention, some studies have reported contradictory results, suggesting that probiotic administration may not always positively impact caries prevention (Aminabadi et al., 2011; Angarita‐Díaz et al., 2020; Cildir et al., 2012; Gizani et al., 2016; Hasslöf et al., 2013; Koopaie et al., 2019; Lin et al., 2017; Nishihara et al., 2014; Pinto et al., 2014; Schwendicke et al., 2014; Stensson et al., 2014; Taipale et al., 2013; Teanpaisan & Piwat, 2014; Villavicencio et al., 2018). While promising, the field of probiotic research faces challenges in clinical trial design and interpretation, as highlighted by recent analyses. These conflicting results underscore the need for more rigorous and standardized research methodologies to elucidate probiotics' efficacy in caries prevention fully (Al‐Madhagi & Alramo, 2023). The probiotic used, how long the intervention lasted, the research methodology, and the target population's characteristics might all contribute to the wide range of findings seen among studies (Amargianitakis et al., 2021; Mokoena et al., 2016). These studies used probiotic bacteria from dairy products, tablets, lozenges, and chewing gum in various dosing regimens to treat dental caries and associated biofilms.

### 3.4 The role of prebiotics and synbiotics in dental caries

In 1995, Gibson and Roberfroid suggested prebiotics to enhance symbiosis among intestinal microorganisms. Prebiotics suppress acidogenic and aciduric microorganisms or improve pH recovery by creating alkali (Gibson & Roberfroid, 1995). Interestingly, certain oral bacteria can utilize prebiotics such as urea and arginine, leading to the synthesis of ammonia and an increase in pH levels. Urea, or carbamide, can be converted into ammonia and bicarbonate ions by bacteria containing the enzyme urease (Amargianitakis et al., 2021; Zaura & Twetman, 2019). Although there has been limited research on the supplementation of urea as a prebiotic for anticaries purposes, studies have shown that it can increase salivary pH and decrease lactic acid production, both contributing to the prevention of caries lesions (Zaura & Twetman, 2019). Arginine, present in protein‐rich diets and salivary polypeptides, can be degraded by the bacterial arginine deiminase system, producing ammonia. This process raises the cytoplasmic and environmental pH levels, providing a health advantage by inhibiting the caries process (Amargianitakis et al., 2021; Zaura & Twetman, 2019). Moreover, the binding of prebiotics to microorganisms' pili prevents their attachment to the host surface, thereby reducing the number of germs present. Prebiotics also enhance the production of lysozyme, an enzyme that penetrates bacterial cell walls through the peptidoglycan layer, effectively killing the bacteria (Agarwal et al., 2022). Non‐digestible fibers such as xylose, xylitol, and arabinose have shown potential as prebiotics that promote beneficial oral microorganism growth (Sato et al., 2017; Valladares‐Diestra et al., 2023). These fibers are not broadly metabolized but elicit a metabolism biased toward health‐promoting microorganisms within the indigenous ecosystem (Bamigbade et al., 2022; Lockyer & Stanner, 2019).

This review emphasized the synbiotic potential for preventing dental caries by mixing the prebiotic with the probiotic (Table 2). The simultaneous administration of prebiotics and probiotics may enhance the ability to inhibit the growth of cariogenic bacteria. However, due to a lack of controlled clinical studies on synbiotics for caries prevention, evidence of their caries‐preventive potential is weak (Amargianitakis et al., 2021; Bijle, Ekambaram, et al., 2020; Bijle, Neelakantan, et al., 2020). A scoping review has demonstrated that synbiotics can reduce caries incidence through mechanisms similar to probiotics. Synbiotics help maintain a high pH in the oral environment, produce antimicrobial substances, compete with pathogenic bacteria for mucosal or binding sites, promote the growth of beneficial oral microorganisms, and modulate the immune response (Amargianitakis et al., 2021; Bijle, Ekambaram, et al., 2020; Bijle, Neelakantan, et al., 2020). Nunpan et al. (2017) studied a synbiotic mixture of galactooligosaccharides as the prebiotic and *L. acidophilus* as the probiotic. Their findings suggest that this synbiotic combination could be therapeutically used to reduce the number of oral *S. mutans*.

**TABLE 2:** Synbiotics have been studied for their effects on dental caries in vitro and in vivo.

References	Subjects/sample	Probiotic	Prebiotic	Suitable probiotic	Suitable prebiotics	Effects
Kojima et al. (2016)	Bacterial cultures	40 *lactobacilli* strains isolated from oral cavity	12 saccharides (Glucose, Maltose, Galactose, Lactose, Xylose, Treharose, Xylitol, Arabinose, Cellobiose, Melezitose, Sucrose, Raffinose)	Five *lactobacilli* strains (*Limosilactobacillus fermentum*, *Lactobacillus plantarum*, *Lactobacillus paracasei*, *L. plantarum*, *Lactobacillus* spp.)	Arabinose, xylose, and xylitol	↓ Growth of *Candida albicans* and *Porphyromonas gingivalis* ↓ Production of insoluble glucan by *Streptococcus mutans* ‐ Oral microbiota
Nunpan et al. (2019)	Bacterial cultures	*S. mutans* and *Lactobacillus acidophilus* ATCC 4356 (ratio of 1:1, 10^6^ cells)	Galactooligosaccharides (1%, 2%, 3%, 4%, and 5% *v*/*v*)		3%, 4%, and 5% of galactooligosaccharides	↓ *S. mutans* growth rate *L. acidophilus* growth rate
			Fructooligosaccharides (1%, 2%, 3%, 4%, and 5% *v*/*v*)		1%, 2%, 3%, 4%, and 5% of fructooligosaccharides	↓ *S. mutans* growth rate *‐ L. acidophilus* growth rate
Nunpan et al. (2017)	Bacterial cultures	*S. mutans* and *L. acidophilus* TISTR 2365T or DSMZ 20079T (ratio of 1:20, 10^7^ cells)	Galactooligosaccharides (1% and 2%)		–	↑ *S. mutans* growth rate ↑ *L. acidophilus* growth rate
			Galactooligosaccharides (3% and 4%)		–	‐ *S. mutans* growth rate ‐ *L. acidophilus* growth rate
Hernández et al. (2020)	Children (5–15 years old, with active dental caries; *n* = 24)	Lactiv® (*L. acidophillus*, *Lactobacillus casei*, *Lactobacillus rhamnosus*, *L. plantarum*, *Bifidobacterium infantis*, and *Streptococcus thermophillus*, *Naturex laboratorios*), for 6 days	Not mentioned			↓ Salivary viscosity ↑ Buffer capacity
Bijle, Ekambaram, et al. (2020), Bijle, Neelakantan, et al. (2020)	Bacterial cultures	*L. rhamnosus* GG	l‐arginine (0.5%, 1%, and 2%)		2% l‐arginine	↑ *L*. *rhamnosus* growth rate ↓ *S. mutans* growth rate ↑ pH ↓ Lactic acid production
Agarwal et al. (2022)	Children (6–10 years old, DMFT score of 5; *n* = 30)	100 g of probiotic yogurt, for 1 month, twice daily	100 g of red banana which contains 40 g of oligosaccharide			↓ Salivary *S. mutans* count ↑ Salivary IgA

Abbreviations: ‐, no effect; ↑, increase; ↓, decrease.

Co‐culturing *S. mutans* with *L. acidophilus* in a medium enriched with galactooligosaccharides or fructooligosaccharides (FOS) significantly reduced the growth of *S. mutans* (Nunpan et al., 2019). However, galactooligosaccharides were ineffective as a prebiotic in a different study and did not enhance the ability of the probiotic *L. acidophilus* to inhibit the development of *S. mutans* (Kojima et al., 2016). In the other research, multispecies probiotics containing *L. acidophilus*, *L. casei*, *L. plantarum*, *L. rhamnosus*, *Bifidobacterium infantis*, and *Streptococcus thermophilus* were used in the study as synbiotic intervention. However, the prebiotic element was not discussed in detail. The researchers observed a significant decrease in salivary viscosity compared to baseline data after 6 days of once‐daily synbiotic administration, while the saliva buffer capacity was enhanced (Hernández et al., 2020). In an in vitro study, the impact of prebiotic l‐arginine and probiotic LGG showed that increasing l‐arginine concentrations promoted the growth of LGG and significantly inhibited the growth of *S. mutans*. Additionally, l‐arginine significantly increased the pH of the medium and reduced the amount of lactic acid produced by LGG biofilms (Bijle, Ekambaram, et al., 2020; Bijle, Neelakantan, et al., 2020). A recent study categorized children aged 6–9 years into three groups: prebiotics, probiotics, and synbiotics. After a month, all three groups showed a significant decrease in *S. mutans*, with no discernible differences. After the intervention, the probiotic and synbiotic groups significantly increased salivary immunoglobulin A (IgA) concentrations (Agarwal et al., 2022).

### 3.5 The role of postbiotics in dental caries

Probiotics can produce metabolites such as biosurfactants, bacteriocins, and EPS to inhibit the adhesion and colonization of cariogenic bacteria. Postbiotics have the potential to provide similar advantages as probiotics while potentially circumventing some of their limitations. Postbiotics may offer similar benefits to probiotics. However, their efficacy has not been conclusively proven or fully characterized. Recent research has shown that postbiotics generated from various microorganisms can inhibit pathogen growth, prevent the formation of biofilms, and kill pathogenic bacteria (Table 3).

**TABLE 3:** Postbiotics have been studied for their effects on dental caries in vitro and in vivo.

References	Probiotic	Postbiotics	Target biofilm	Suitable probiotic	Effects
OmerOglou et al. (2022)	*Lactiplantibacillus plantarum* EIR/IF‐1 *Lactiplantibacillus curvatus* EIR/DG‐1 *L. curvatus* EIR/BG‐2	Cell‐free supernatants	*Streptococcus mutans*	*L. plantarum* EIR/IF‐1	↓ *S. mutans* growth rate ↓ Biofilm formation of *S. mutans* ↓ Expression of gtfC, comA, and comX
Ahn et al. (2018)	*L. plantarum*	Lipoteichoic acid	*S. mutans* KCTC 3065	–	↓ Biofilm formation of *S. mutans* ↓ Exopolysaccharide production
Jeong et al. (2018)	*Lactobacillus kefiranofaciens* DD2 *L. plantarum* ATCC 10012 *Lactobacillus johnsonii* JCM 1022 *Lactobacillus rhamnosus* ATCC 7469	Culture supernatant	*S. mutans* and *Streptococcus sobrinus*		Inhibited growth of *S. mutans* and *S. sobrinus* ↓ Biofilm formation of *S. mutans* and *S. sobrinus* ↓ Expression of ftf, gtfB, gtfC, brpA, comDE, vicR, gbpB and spaP (by *L. kefiranofaciens* DD2)
Kim et al. (2019)	*L. plantarum*	Lipoteichoic acid	*Actinomyces naeslundii*, *Enterococcus faecalis*, *L. salivarius*, and *S. mutans* KCTC 3065	–	↓ Biofilm formation
Kim et al. (2022)	Six *Lactobacillus brevis* Strains	Cell‐free supernatants	*S. mutans* KCTC 5124 *S. mutans* KCTC 5458 *S. mutans* KCTC 5316	*L. brevis* KCCM 202399	High antimicrobial activity ↓ Biofilm formation
Ciandrini et al. (2016)	*L. reuteri* DSM 17938, *Lactobacillus acidophilus* DDS‐1, *L. rhamnosus* ATCC 53103, and *Lactobacillus paracasei* B21060	Biosurfactants	*S. mutans* ATCC 25175 *S. oralis* ATCC 9811	*L*. *reuteri* DSM 17938	↓ *S. mutans* growth rate ↓ *S. oralis* growth rate ↓ Biofilm formation of *S. mutants* and *S. oralis*
Savabi et al. (2014)	*Lactobacillus casei* ATCC39392	Biosurfactants	*S. mutans ATCC35668*	–	↓ *gftB*/*C* and *tft* gene expression
Salehi et al. (2014)	*L. reuteri* DSM20016	Biosurfactants	*S. mutans ATCC35668*	–	↓ *gftB*/*C* and *tft* gene expression
Tahmourespour et al. (2019)	*L. rhamnosus* ATCC7469	Biosurfactants	*S. mutans ATCC35668* *S. mutans* 22	–	↓ Biofilm formation of *S. mutants* ↓ *gftB*/*C* and *tft* gene expression
Rossoni, de Barros, et al. (2018), Rossoni, dos Santos Velloso, et al. (2018)	22 strains of *Lactobacillus* (*L. paracasei*, *L. fermentum* and *L. rhamnosus*)	Cell‐free supernatant	*S. mutans* UA159	*L. paracasei* 11.6, *L. paracasei* 25.4, *L. fermentum* 20.4 and *L. paracasei* 20.3	↓ *S. mutans* growth rate ↓ *S. mutans* count ‐ pH
Srivastava et al. (2020)	*Lactobacillus plantarum* 108	Cell‐free supernatant	*S. mutans* UA159 *C. albicans SC5314*	–	↓ Growth of *S. mutans* and *C. albicans* ↓ Biofilm formation of *S. mutants* and *C. albicans* ↓ GtfB, gtfC and gtfD gene expression in *S. mutans* biofilms ↓ HWP1, ALS1 and ALS3 gene expression in *C. albicans* biofilms
Lin et al. (2015)	*L. casei* Shirota, *L. casei* LC01, *L. plantarum* ST‐III, *L. paracasei* Lpc‐37, and *L. rhamnosus* HN001	Cell‐free supernatant	*S. mutans* UA159	*L. casei* Shirota and *L. rhamnosus* HN001	↓ Growth of *S. mutans* ↓ Biofilm formation of *S. mutans*
Jeong et al. (2018)	*L. kefiranofaciens* DD2, DD5, and DD6 *L. plantarum* ATCC 10,012, *L. johnsonii* JCM 1022, and *L. rhamnosus* ATCC 7469	Cell‐free supernatant	*S. mutans* *S. sobrinus*	*L. kefiranofaciens* DD2	↓ Growth of *S. mutans* and *S. sobrinus* ↓ Biofilm formation of *S. mutans* and *S. sobrinus*
Rossoni, de Barros, et al. (2018), Rossoni, dos Santos Velloso, et al. (2018)	Thirty *Lactobacillus* strains	Cell‐free supernatant	*C. albicans*	*L*. *paracasei* 28.4, *L*. *rhamnosus* 5.2 and *L*. *fermentum* 20.4	↓ Biofilm formation of *C. albicans* ↓ *C. albicans* count ↓ Expression of ALS3, HWP1, EFG1 and CPH1
Wasfi et al. (2018)	*Lactobacillus casei* ATCC 393, *L. reuteri* ATCC 23272, *L. plantarum* ATCC 14917 and *L. salivarius* ATCC 11741	Cell‐free supernatant	*S. mutans* ATCC 25175	–	↓ Growth of *S. mutans* ↓ Biofilm formation of *S. mutans* ↓ Expression of atpD and aguD ↓ Expression of gtfBCD and sacB ↓ Expression of vicKR and comCD ↑ IFN‐γ production ↓ IL‐10 production
Chen, Daliri, et al. (2020), Chen, Schlafer, et al. (2020)	*L. reuteri* ATCC PTA 5289 *Streptococcus oligofermentans* DSM 8249	Cell‐free supernatant	*S. mutans* *L. rhamnosus Actinomyces naeslundii*		↓ Viable bacteria ↓ *S. mutans* and *A. naeslundii* counts ‐ *L. rhamnosus* counts ‐ pH ↓ Mineral Loss of Enamel Lesions
Song and Lee (2017)	*L. acidophilus* ATCC 4356, *L. casei* ATCC 334, *L. rhamnosus* GG (ATCC 53103), and *Bifidobacterium breve* ATCC 15700	Cell‐free supernatant	*C. albicans* ATCC 10231	*L. rhamnosus* and *L. casei*	↓ Growth of *C. albicans* ↓ Biofilm formation of *C. albicans*
Yang et al. (2021)	*L. reuteri AN417*	Cell‐free supernatant	*S. mutans* KCTC 3065 *P. gingivalis* BAA‐308 *F. nucleatum*	40% (v/v) Cell‐free supernatant	↓ Growth of *S. mutans* ↓ Growth of *P. gingivalis* ↓ Growth of *F. nucleatum* ↓ Viability of oral pathogenic bacteria ↓ Biofilm formation of *P. gingivalis* and *S. mutans* ↓ Expression of rgpA, rgpB, hagA, hagB, and kgp
Jung et al. (2021)	Eight probiotic strains (*L. plantarum* MG207, *L. paracasei* MG310, *L. casei* MG311, *L. rhamnosus* MG316, *L. salivarius* MG4265, *L. lactis* MG5125, *L. fermentum* MG901, and *L. plantarum* MG989)	Cell‐free supernatant	*S. mutans* KCTC3065	*L. salivarius* MG4265	↓ Growth of *S. mutans* ↓ Biofilm formation of *S. mutans*
Banakar et al. (2023)	*L. rhamnosus GG* (LGG) *L. reuteri* (LR)	Probiotic metabolites	*S. mutans*		↓ *S. mutans* (Only LGG) ↓ *S. mutans* metabolic activity ↓ Expression of gtfB

Abbreviations: ‐, no effect; ↑, increase; ↓, decrease; gtfC, glucan forming gene.

Jeong et al. (2018) evaluated the mRNA levels of *S. mutans* genes encoding virulence proteins related to carbohydrate metabolism (*ftf*, *gtfB*, and *gtfC*), biofilm formation (*brpA*, *comDE*, and *vicR*), and adhesion (*gbpB* and *spaP*) by reverse transcription real‐time PCR. They found that *kefiranofaciens* DD2 metabolites potentially downregulate the expression of these virulence genes. *Lactobacillus* sp. cell‐free supernatant inhibits *S. mutans* growth through various mechanisms, including the production of organic acids and peroxide, prevention of cell aggregation and biofilm formation, and downregulation of virulence genes such as acid tolerance genes (*atpD* and *aguD*), exopolysaccharide‐producing genes (*gtfBCD* and *sacB*), and quorum‐sensing genes (*vicKR* and *comCD*) (Wasfi et al., 2018). Additionally, it exerts an immunomodulatory impact by increasing IFN expression and decreasing IL‐10 production (Wasfi et al., 2018). OmerOglou et al. (2022) found that all postbiotics (*Cell‐free supernatants*) derived from *Lactiplantibacillus* spp. were beneficial in reducing the growth of *S. mutans*, mostly via organic acid synthesis and in decreasing cariogenic biofilm formation via repression of pathogen quorum‐sensing‐mediated virulence genes. Lipoteichoic acid isolated from *L. plantarum* has been shown to suppress the production of *S. mutans* biofilms (Ahn et al., 2018) or oral multispecies biofilms (Kim et al., 2019) and might be used to create potent anticaries agents. Research also shows that *Lactobacillus* spp. biosurfactants significantly reduce *S. mutans* and *S. oralis* biofilm development and growth (Ciandrini et al., 2016). These biosurfactants effectively inhibit the expression of adhesive‐promoting genes (*gtfB*/*C* and *tft*), which are crucial for the adhesion of *S. mutans* during biofilm formation (Salehi et al., 2014; Savabi et al., 2014; Tahmourespour et al., 2019). Most *Lactobacillus* spp. also show antimicrobial effects on *S. mutans* in their cell‐free supernatant (Banakar et al., 2023; Jung et al., 2021; Kim et al., 2022; Rossoni, de Barros, et al., 2018; Rossoni, dos Santos Velloso, et al., 2018).

Some studies pointed to the role of *Candida albicans* in creating and maintaining cariogenic biofilm (James et al., 2016; Kim et al., 2017; Li et al., 2023). James et al. (2016) have seen that the expression of critical genes related to biofilm formation, host cell invasion, and virulence in *C. albicans*, including *ALS3*, *EFG1*, *SAP5*, and *HWP1*, were downregulated by the cell‐free supernatant of *L. plantarum*, *L. helveticus*, and *S. salivarius*. Furthermore, *Lactobacillus* spp. have been demonstrated to produce acids or exometabolites that inhibit *C. albicans* growth (Song & Lee, 2017) and are linked to the downregulation of the *ALS3*, *HWP1*, *CPH1*, and *EFG1* genes (Rossoni, de Barros, et al., 2018; Rossoni, dos Santos Velloso, et al., 2018). Srivastava et al. (2020) found that the cell‐free supernatant obtained from *L. plantarum* significantly downregulated the expression of hyphal development‐related genes in *C. albicans* (*HWP1*, *ALS1*, and *ALS3*) and glucosyltransferases (*gtfB*, *gtfC*, and *gtfD*) in *S. mutans* biofilms. Although most studies confirm the specific role of postbiotics similar to probiotics, Chen et al. reported that viable probiotics *L. reuteri* and *Streptococcus oligofermentans* could suppress the cariogenic effects of multispecies. However, their cell‐free supernatant was ineffective (Chen, Daliri, et al., 2020; Chen, Schlafer, et al., 2020).

## 4 DISCUSSION

This scoping review aimed to investigate the role of probiotics, synbiotics, and postbiotics on dental caries and cariogenic bacteria by focusing on new findings. Among the 42 articles reviewed, only eight studies reported no effect or an increase in caries lesions, while most indicated that probiotics reduce the risk of caries. Twenty‐two studies examined the impact of probiotics on caries‐associated bacteria, such as *S. mutans* and *lactobacilli*. The effects of probiotic supplementation on *S. mutans* and *lactobacilli* levels have yielded conflicting results, and not all probiotic therapies have shown improvements in caries prevention. Specific probiotic strains have been shown to reduce *S. mutans* effectively in saliva and/or plaque in 23 articles and may prevent dental caries (Alamoudi et al., 2018; Burton, Drummond, et al., 2013; Burton, Wescombe, et al., 2013; Campus et al., 2014; Cannon et al., 2013; Di Pierro et al., 2015; Ferrer et al., 2020; Ghasemi et al., 2017; Jose et al., 2013; Juneja & Kakade, 2012; Kaur et al., 2018; Lai et al., 2021; Lin & Pan, 2014; Mahantesha et al., 2015; Manmontri et al., 2020; Pahumunto et al., 2018; Saha et al., 2014; Sudha et al., 2020; Teanpaisan & Piwat, 2014; Wasfi et al., 2018; Yadav et al., 2015; Zare et al., 2015). Conversely, 12 studies suggest that probiotics do not significantly affect *S. mutans* counts, indicating that probiotic administration may not positively impact caries prevention (Aminabadi et al., 2011; Angarita‐Díaz et al., 2020; Cildir et al., 2012; Gizani et al., 2016; Hasslöf et al., 2013; Koopaie et al., 2019; Lin et al., 2017; Nishihara et al., 2014; Pinto et al., 2014; Schwendicke et al., 2014; Stensson et al., 2014; Villavicencio et al., 2018). Among the 15 studies that evaluated the effect of probiotics on *lactobacilli* counts, seven reported a reduction in *lactobacilli* (Alamoudi et al., 2018; Cannon et al., 2013; Gizani et al., 2016; Javid et al., 2020; Sudha et al., 2020; Villavicencio et al., 2018; Zare et al., 2015). Meanwhile, the remaining eight studies found no effect or increased *lactobacilli* count in saliva and/or plaque (Cildir et al., 2012; Hasslöf et al., 2013; Lin et al., 2017; Manmontri et al., 2020; Nishihara et al., 2014; Pinto et al., 2014; Stensson et al., 2014; Teanpaisan & Piwat, 2014). The difference in the results of studies on the effectiveness of probiotics in reducing the levels of *S. mutans* and *Lactobacillus* can be attributed to the type of probiotic used, environmental factors, the combination of probiotics with other substances, individual differences, the method of administration and Inconsistencies between trials (Amargianitakis et al., 2021; Mahasneh & Mahasneh, 2017; Saïz et al., 2021).

Although the literature describes several mechanisms of action for probiotics, many of these processes remain unclear. Possible pathways for the positive effects of probiotics are described, including interaction with other microbes in the biofilm to eliminate or restrict pathogens, co‐aggregation, competitive inhibition, production of hydrogen peroxide, organic acids, and bacteriocin‐like compounds, as well as immunological effects on the mucosa, including stimulation of macrophage activity and phagocytosis (Allaker & Stephen, 2017; Devine et al., 2015; Lin et al., 2018; Shakib et al., 2020). Probiotics have been shown to inhibit cariogenic acidogenic and aciduric bacteria. However, it is important to consider that biofilm formation and acid production are risk factors that may influence the effects of probiotic strains on caries risk (Allaker & Stephen, 2017; Amargianitakis et al., 2021; Cagetti et al., 2013). Seven studies have demonstrated increased pH and probiotics' cariostatic effects on oral biofilm's acidogenicity (Campus et al., 2014; Di Pierro, 2016; Ferrer et al., 2020; Lai et al., 2021; Lin et al., 2017; López‐López et al., 2017; Saha et al., 2014). However, five studies have reported contradictory results, indicating that the administration of probiotics does not affect oral pH (Koopaie et al., 2019; Nishihara et al., 2014; Sakhare et al., 2021; Sudha et al., 2020; Villavicencio et al., 2018). Given the existing contradictions, further studies are needed to investigate the mechanisms of action of probiotics in caries and oral diseases, particularly their effects on immunoglobulins.

The stability of the oral microbiome significantly influences the effectiveness of probiotics. If the oral microbiota remains steady, it may exhibit resistance to alterations caused by probiotics, hence diminishing the effectiveness of the probiotics. Conversely, if the oral microbiota is unstable, it could be more prone to alterations caused by probiotics, thereby enhancing the effectiveness of the probiotics (Rad et al., 2023; Radaic & Kapila, 2021). The oral microbiome of children exhibits greater susceptibility to environmental influences than adults (Wei et al., 2021). Nevertheless, it is crucial to acknowledge that the efficacy of probiotics can significantly differ based on the individual and the particular strains of probiotics employed (Amargianitakis et al., 2021). Hence, although the stability of a child's oral microbiota can impact the efficacy of probiotics, it is not the sole determinant in assessing the suitability of probiotics for a specific individual (Amargianitakis et al., 2021). Yli‐Knuuttila et al. (2006) showed that LGG could not colonize the oral cavity of young adults. However, they suggested that permanent colonization might be possible if the bacteria are administered to children at a young age. According to Devine and Marsh (2009), this discrepancy might be attributed to the variability of the child's local microbiota. Nevertheless, it is crucial to acknowledge that although probiotics might have advantageous effects, they must be employed cautiously, particularly when administered to children. Certain probiotic products recommended for children may have a high sugar content, which can potentially lead to illnesses such as diabetes, dental erosion, and dental caries (Mantegazza et al., 2018).

One issue is the challenge exogenous probiotic bacteria pose in establishing colonization within the preexisting oral microbiota. In order to achieve optimal efficacy, a probiotic strain must exhibit strong adhesion to dental surfaces. *Lactobacilli*, in particular, have poor tooth adhesion, raising concerns about long‐term stability. Limited data are available on the concentration of probiotics in saliva and their persistence on tooth structure (Amargianitakis et al., 2021). Meurman et al. (1994) found that salivary counts of LGG decreased 2 weeks after discontinuing consumption of probiotic yogurt containing LGG, indicating that temporary colonization was not achieved, even in individuals with undetectable levels of *lactobacilli*, suggesting unsuitable environmental conditions for *lactobacilli* growth in the oral cavity of the subjects. Aminabadi et al. (2011) conducted a study to determine if the beneficial effects of chlorhexidine (CHX) in managing the oral microbiota could enhance the effect of LGG. The researchers concluded that CHX increased the stability of LGG oral colonization for at least 5 weeks after treatment cessation. Since probiotic bacteria cannot permanently colonize the oral cavity, they must be consumed daily. Therefore, incorporating probiotics into daily health products such as dairy products could be a viable approach to ensure consistent delivery (da Cruz et al., 2022). Long‐term probiotic therapy has also been demonstrated to reduce the number of cariogenic bacteria, which may help prevent caries even though there was no such impact in short‐term administration (Sakhare et al., 2021). Nevertheless, some studies have indicated a decrease in the beneficial effects of probiotics on reducing dental caries as the study period extends (Ashwin et al., 2015; Mahantesha et al., 2015).

Probiotics can be delivered by dairy food (milk, cheese, yogurt, curd, gum, and ice cream) (Aminabadi et al., 2011; Angarita‐Díaz et al., 2020; Ashwin et al., 2015; Banakar et al., 2022; da Cruz et al., 2022; Ghasemi et al., 2017; Jose et al., 2013; Juneja & Kakade, 2012; Lin et al., 2017; Mahantesha et al., 2015; Manmontri et al., 2020; Pahumunto et al., 2018; Pinto et al., 2014; Rodríguez et al., 2016; Sakhare et al., 2021; Teanpaisan & Piwat, 2014; Villavicencio et al., 2018; Yadav et al., 2015; Zare et al., 2015). These products have naturally high calcium and phosphate content, which helps remineralize hard dental tissues and prevents the production of cariogenic bacteria acid (Cagetti et al., 2013). Individuals with dairy allergies may consider using alternate probiotic options such as capsules, liquid forms, tablets, drops, lozenges, sweetened cakes, and gums. Notably, one study employed chewing gums as a delivery vehicle (Kaur et al., 2018). Sugar‐free chewing gum can also positively impact dental health by increasing saliva production, reducing plaque acidogenicity, and minimizing enamel demineralization (Banakar et al., 2022; Cagetti et al., 2013). In the remaining studies (16 studies), probiotics were delivered through various products such as lozenges, tablets, drops, and powders (Alamoudi et al., 2018; Burton, Drummond, et al., 2013; Burton, Wescombe, et al., 2013; Campus et al., 2014; Cannon et al., 2013; Cildir et al., 2012; Di Pierro, 2016; Di Pierro et al., 2015; Ferrer et al., 2020; Gizani et al., 2016; Hedayati‐Hajikand et al., 2015; Koopaie et al., 2019; Lai et al., 2021; Laleman et al., 2015; Nishihara et al., 2014; Stensson et al., 2014; Sudha et al., 2020; Taipale et al., 2013). Additionally, three studies utilized cereals or plant‐based milk, such as soy milk and cake, as delivery vehicles (Hasslöf et al., 2013; Koopaie et al., 2019; Lin & Pan, 2014).

Research is now being conducted to investigate the influence of probiotic quantity and dosage on the development of dental caries. According to Amargianitakis et al.'s (2021) review, the efficacy of probiotics in preventing caries may be influenced by the dosage and duration of their administration. Nevertheless, the literature lacks in‐depth discussions regarding the precise dosages of probiotics and their direct influence on the occurrence and development of dental caries. Consequently, there is currently no definitive recommendation in the existing research regarding the most effective dosage of probiotics for preventing dental caries (Saïz et al., 2021). The efficacy of probiotics also depends on an adequate quantity of viable probiotic cells reaching the caries surface to exert their effects. However, the viability and survival rate of probiotic bacteria varies among strains, and many probiotics may degrade in products due to exposure to low pH and oxygen during chilling, transportation, and storage (Bajaj et al., 2021; Evivie et al., 2017). Moreover, ensuring probiotics' viability and effective delivery to the oral cavity remains a significant challenge. Encapsulation of probiotic bacteria in protective materials like alginate and carrageenan has significantly improved their survival in food products. This strategy may also enhance their persistence in the oral environment and provide sustained oral health benefits (Afzaal et al., 2019). Therefore, developing micro‐ and nano‐sized drug delivery systems encapsulating probiotics may offer some protection against environmental stresses (Evivie et al., 2017; Gyawali et al., 2023). Further rigorous clinical trials are required to ascertain the optimal probiotic combinations, the suitable probiotic delivery method, and the frequency and dosage of probiotic administration (Amargianitakis et al., 2021; Saïz et al., 2021).

In healthy individuals, side effects from oral probiotics are unlikely severe, typically mild, and digestive. However, in individuals with preexisting immunodeficiency conditions, there is a potential risk of systemic infections that may require antimicrobial treatment (Devine & Marsh, 2009). It is important to note that the mechanisms of action of probiotics in the oral environment are non‐specific and not solely focused on pathogenic bacteria (Chopra & Mathur, 2013; Ramanujam et al., 2019). There are several unanswered questions regarding using probiotics in the oral cavity. For instance, probiotics' long‐term safety and efficacy in the oral cavity have not been thoroughly investigated. Moreover, the optimal strains, dosages, and delivery methods for probiotics in oral health applications need to be determined.

Prebiotics are fermentable substances that influence the balance of microflora by providing nutrition and stimulating probiotic development and activity. Urea (carbamide) and arginine have been investigated as prebiotics for controlling dental caries. Urea supplementation has been associated with calculus development, and limited evidence supports its preventive effects on dental caries (Amargianitakis et al., 2021; Zaura & Twetman, 2019). On the other hand, the amino acid arginine has shown prebiotic properties that may help reduce dental caries (Zaura & Twetman, 2019). Our findings suggest that synbiotics may be a promising approach to reducing cariogenic bacteria, as they can modulate the oral microbiota and improve the balance between beneficial and harmful bacteria. Synbiotics can modulate the immune response, promote beneficial bacteria growth, and inhibit oral pathogenic bacteria growth (Amargianitakis et al., 2021; Bijle, Ekambaram, et al., 2020; Bijle, Neelakantan, et al., 2020). However, there is a lack of information on how synbiotics, similar to probiotics, can effectively prevent caries. Several studies have demonstrated that synbiotic administration can reduce the growth of cariogenic pathogens, particularly *S. mutans*, by reducing lactic acid production, increasing pH, and stimulating salivary IgA production (Agarwal et al., 2022; Bijle, Ekambaram, et al., 2020; Bijle, Neelakantan, et al., 2020; Hernández et al., 2020; Kojima et al., 2016; Nunpan et al., 2017, 2019).

Further research is needed to learn more about the best combination of prebiotics and probiotics (symbiotic) and the action of synbiotics against the cariogenic pathogen. Studies on synbiotics and dental caries are preclinical, and only a few clinical studies are available. Furthermore, the optimal combination and dosage, frequency, and duration of synbiotic supplementation for dental caries prevention remain to be determined. Future clinical trials are needed to determine the optimal synbiotic formulation, dosage, and regimen for dental caries prevention.

**FIGURE 6:**
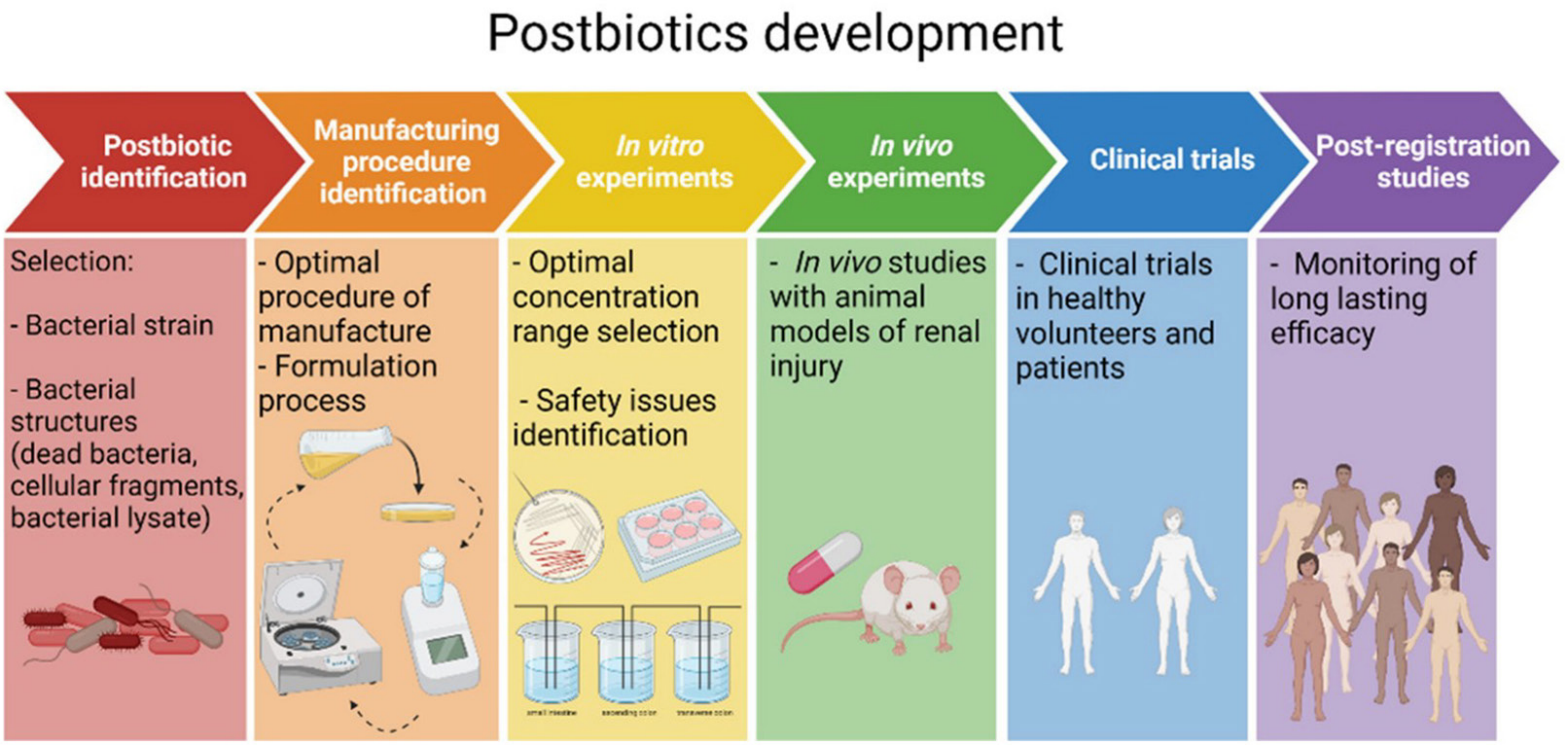
A roadmap toward the postbiotic therapy (Favero et al., 2022).

This study also investigates the effect of postbiotics on cariogenic bacteria, which has been less explored in previous reviews. Different postbiotic types, including cell‐free supernatants, secreted proteins, bacteriocins, biosurfactants, and cell wall molecules, have been studied and shown promising results in preventing biofilm formation and caries. Postbiotics prevent the growth of cariogenic bacteria through mechanisms such as maintaining an acidic pH, antimicrobial effect through antimicrobial proteins, organic acids, biosurfactants, fatty acids, hydroxyl radicals, and bacteriocins, as well as enzymatic degradation of biofilms, and genes modification. The review findings suggest that postbiotics may act as anti‐biofilm agents against various microorganisms. They may either suppress biofilm formation or disrupt existing biofilms. Postbiotics can also induce immune‐modulating, antibacterial, and anti‐inflammatory responses, making them valuable agents for combating biofilms (Ahn et al., 2018; Banakar et al., 2023; Chen, Daliri, et al., 2020; Chen, Schlafer, et al., 2020; Ciandrini et al., 2016; Jeong et al., 2018; Jung et al., 2021; Kim et al., 2019; Lin et al., 2015; OmerOglou et al., 2022; Rossoni, de Barros, et al., 2018; Rossoni, dos Santos Velloso, et al., 2018; Salehi et al., 2014; Savabi et al., 2014; Song & Lee, 2017; Srivastava et al., 2020; Tahmourespour et al., 2019; Yang et al., 2021). Furthermore, using postbiotics instead of probiotics in immunocompromised patients may be a valuable option for reducing the risk of infection. However, there is still a need for further research and clinical trials to fully evaluate the therapeutic potential of postbiotics in dental caries prevention and treatment. Figure 6 shows the roadmap toward postbiotic therapy. This roadmap illustrates the sequential progression of postbiotic development, starting from the initial identification of postbiotics and concluding with the final post‐registration investigations. For preparing postbiotics, aspects need to be considered at the industrial level, such as fermentation media, bacterial proliferation and concentration procedures, downstream processing, quality control measures, and standardization of the final product. Additionally, factors such as scalability, cost‐effectiveness, and regulatory compliance must be considered to ensure successful postbiotic commercial production (Asif et al., 2023).

There were limitations in this study. There was heterogeneity in the methodologies used across the included studies. The input studies varied in their design (in vitro vs clinical trials), population characteristics, probiotic strains, dosage regimens, delivery methods, and duration of interventions. This made it difficult to compare the results directly and draw definitive conclusions. Also, clinical trial studies have been fewer, especially on synbiotics and postbiotics. More randomized controlled clinical trials are needed to validate the findings from preclinical research and establish the efficacy of synbiotics and postbiotics for caries prevention. The mechanisms of action of biotics remain not fully understood. While potential pathways have been proposed, further research is still required to elucidate the specific molecular and cellular mechanisms involved. In this study, we tried to reduce bias as much as possible with a specific protocol and review of most sources. However, this scoping review aimed to map the existing literature but did not perform quality assessments of the included studies. As such, the findings may be subject to bias inherent to individual studies. Future systematic reviews and meta‐analyses examining specific probiotic strains or combinations could provide better estimates of effectiveness by incorporating risk of bias assessments.

In conclusion, most studies examined in this scoping review indicate that biotics can be supportive in reducing cariogenic bacteria. While they cannot substitute primary preventive measures such as toothbrushing and flossing to prevent dental caries, biotics may be a valuable adjunct therapy. It is crucial to recognize that the efficacy of probiotics is primarily influenced by the specific strain employed. Factors such as the choice of a potential probiotic strain, optimal dosage, duration of treatment, delivery method, and interaction with the host all contribute to the positive outcomes of probiotic supplementation. This review highlights the importance of selecting robust probiotics that can reduce caries‐associated bacterial populations, generate antimicrobial compounds, and elevate oral pH levels. The stability of children's oral microbiota makes them an ideal target population for probiotic interventions compared to adults. To maintain their effectiveness, probiotics should be consumed daily. Incorporating probiotics into everyday preventive health products, such as dairy items, maybe one approach to ensuring consistent administration. Combining probiotics and prebiotics, known as synbiotics, is a promising strategy for preventing and managing caries. Furthermore, this review draws attention to the potential benefits of postbiotics in reducing cariogenic bacteria. By shifting the focus from live bacteria to bacteria‐derived compounds, postbiotics may offer a safer alternative, even for immunocompromised patients, while still exerting beneficial effects on the host. Due to the limited knowledge regarding the application of postbiotics in caries prevention, future clinical studies are recommended to examine their effectiveness and the mechanisms by which they exert their protective effects against dental caries.

## AUTHOR CONTRIBUTIONS

**Morteza Banakar:** Conceptualization (equal); data curation (equal); formal analysis (equal); funding acquisition (equal); investigation (equal); methodology (equal); software (equal); validation (equal); visualization (equal); writing – original draft (equal); writing – review and editing (equal). **Gustavo Vicentis Oliveira Fernandes:** Investigation (equal); methodology (equal); validation (equal); writing – original draft (equal); writing – review and editing (equal). **Shahroo Etemad‐Moghadam:** Conceptualization (equal); data curation (equal); formal analysis (equal); investigation (equal); validation (equal); visualization (equal); writing – original draft (equal); writing – review and editing (equal). **Roland Frankenberger:** Conceptualization (equal); data curation (equal); formal analysis (equal); investigation (equal); methodology (equal); validation (equal); visualization (equal); writing – original draft (equal); writing – review and editing (equal). **Maryam Pourhajibagher:** Conceptualization (equal); data curation (equal); formal analysis (equal); investigation (equal); methodology (equal); visualization (equal); writing – original draft (equal); writing – review and editing (equal). **Majid Mehran:** Conceptualization (equal); data curation (equal); investigation (equal); methodology (equal); validation (equal); visualization (equal); writing – original draft (equal); writing – review and editing (equal). **Mohammad Hossein Yazdi:** Conceptualization (equal); data curation (equal); formal analysis (equal); investigation (equal); methodology (equal); validation (equal); visualization (equal); writing – original draft (equal); writing – review and editing (equal). **Roza Haghgoo:** Conceptualization (equal); data curation (equal); investigation (equal); methodology (equal); resources (equal); software (equal); validation (equal); visualization (equal); writing – original draft (equal); writing – review and editing (equal). **Mojgan Alaeddini:** Conceptualization (equal); funding acquisition (equal); investigation (equal); methodology (equal); project administration (equal); software (equal); supervision (equal); visualization (equal); writing – original draft (equal); writing – review and editing (equal).

## CONFLICT OF INTEREST STATEMENT

The authors declare no competing interests.

## Data Availability

All data are available within the article.
